# Refractory Thrombocytopenia in a 29-Year-Old Pregnant Woman With Autoimmune Hepatitis: A Case Report and Literature Review

**DOI:** 10.7759/cureus.49189

**Published:** 2023-11-21

**Authors:** Hythem A Al-Sum, Suhad M Alsurori, Maha N Alkhlassi, Albandari A Alanazi, Ibtisam N Alkhlassi, Salma N Alkhlassi

**Affiliations:** 1 Department of Obstetrics and Gynaecology, Division of Maternal Fetal Medicine, King Abdulaziz Medical City, Ministry of National Guard Health Affairs, Riyadh, SAU; 2 College of Medicine, King Saud Bin Abdulaziz University for Health Sciences, Riyadh, SAU; 3 College of Medicine, King Saud University, Riyadh , SAU; 4 College of Medicine, King Saud University, Riyadh, SAU

**Keywords:** aih, autoimmune hepatitis in pregnancy, thrombocytopenia in pregnancy, refractory thrombocytopenia, aih -autoimmune hepatitis, autoimmune hepatitis with cirrhosis

## Abstract

Autoimmune hepatitis (AIH) is a rare autoimmune liver disease that mostly affects women in their reproductive years, leading to impaired fertility. Nonetheless, the majority of women with well-controlled AIH have a favorable prognosis for pregnancy. This case report describes a 29-year-old pregnant woman with cirrhosis secondary to AIH who presented with severe thrombocytopenia. Her labs showed a decline in her platelet counts from 28 × 10^9^/L before pregnancy to 20 × 10^9^/L during pregnancy. Her abdominal ultrasound showed liver cirrhosis secondary to AIH and splenomegaly. Throughout pregnancy, various scans were performed to monitor the fetal well-being, which showed normal results. She was on a medication regimen that included nadolol of 80 mg/kg/day, prednisolone of 5 mg/kg/day, and azathioprine of 50 mg/kg/day. Due to a breech presentation, the patient was scheduled for a cesarean section. She received two courses of dexamethasone at 20 mg/day for four days within two weeks of delivery. On the day of her scheduled C-section, tranexamic acid of 1 g TID for two days was administered, and she received platelet transfusions of 12 units both before and after the procedure, with an additional 6 units administered during the procedure. Despite proper management, her platelet count remained consistently low. However, she successfully delivered a healthy baby, and the overall condition of the patient was stable.

## Introduction

Autoimmune hepatitis (AIH) is a rare autoimmune liver disease that primarily affects women during their reproductive years, leading to amenorrhea and infertility. However, when the disease is well-controlled, most women with AIH have a positive prognosis for pregnancy [1]. The underlying pathology of AIH involves a gradual inflammation of hepatocytes, which cannot be attributed to common causes of chronic liver disease such as alcohol-related liver disease, viral hepatitis, exposure to hepatotoxic substances, or hereditary liver disorders [2-6]. Various clinical studies have examined pregnancy outcomes in AIH patients [6-11]. Some studies suggest that flares of the disease are more common in the postpartum period compared to during pregnancy [6,8,9,11], while others report remission of the disease during pregnancy [9].

Here, we describe a case of severe and refractory thrombocytopenia in a pregnant woman with autoimmune hepatitis despite receiving proper treatment and present the outcomes and management of AIH in pregnancy.

## Case presentation

A 29-year-old pregnant woman is in the 38th week of gestation. She is identified as gravida 2 para 1 plus 0, with a history of liver cirrhosis secondary to AIH. Throughout her pregnancy, her hemoglobin level remained stable at 97 g/L, both before and during pregnancy. However, her platelet count exhibited a decrease from 28 × 10^9^/L before pregnancy to 20 × 10^9^/L during pregnancy. To manage her medical condition and support her pregnancy, the patient was prescribed a medication regimen that included nadolol at a dosage of 80 mg taken once daily, prednisolone at a dosage of 5 mg taken once daily, and azathioprine at a dosage of 50 mg taken once daily.

**Figure 1:**
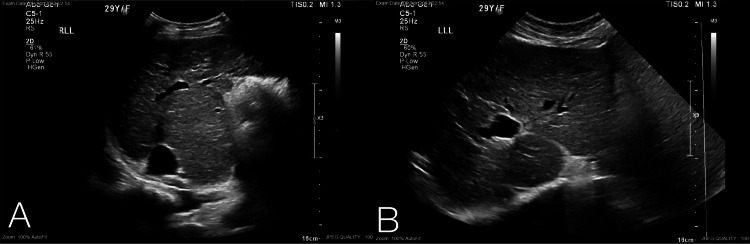
Right and left lower lobe of the liver showing cirrhosis without clear focal lesion.

**Figure 2:**
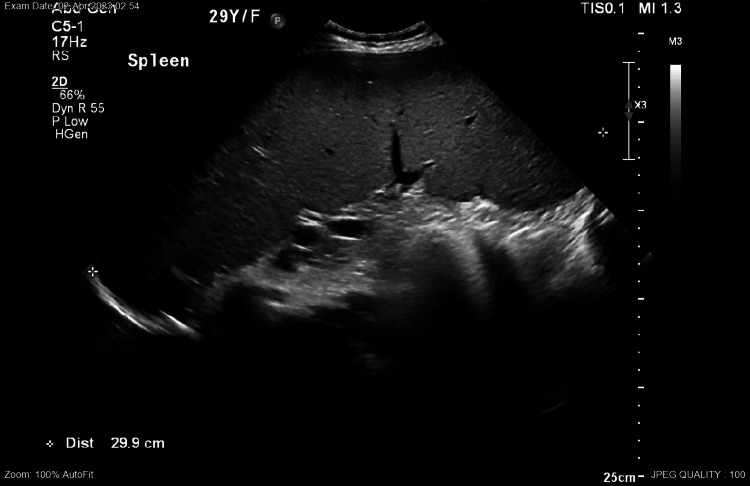
Splenomegaly measuring 29 cm × 11 cm.

**Figure 3:**
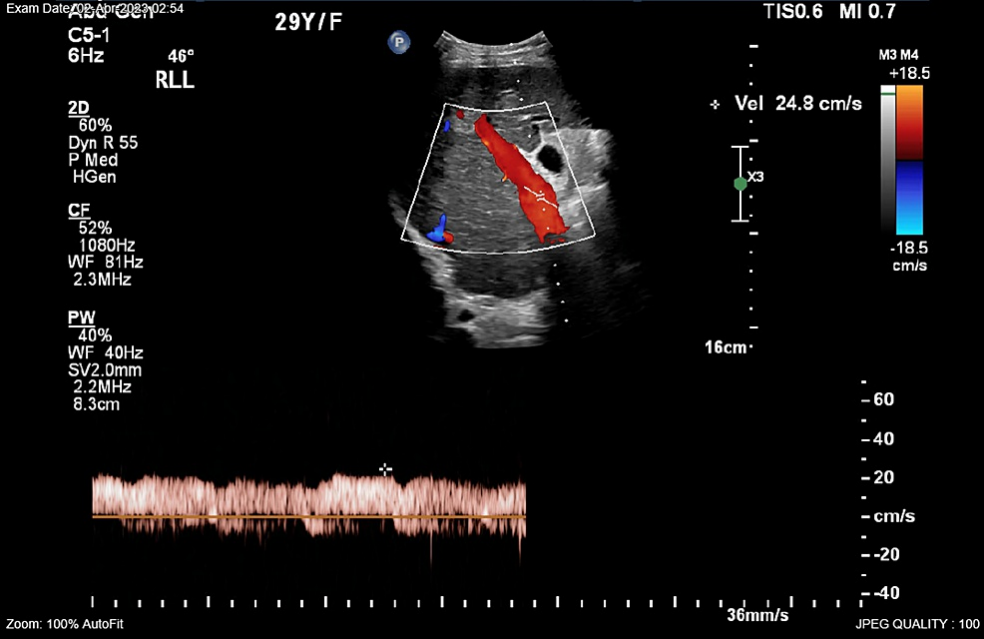
Patent portal vein with normal waveform, velocity, and direction.

During pregnancy, abdominal ultrasound was done, showing liver cirrhosis (Figure 1A-1B), splenomegaly (Figure 2), and normal portal vein doppler (Figure 3).

Various scans were performed to monitor the well-being of the fetus. A nuchal translucency scan (NT) was conducted, which revealed normal thickness. A fetal anatomy scan was conducted at 21 weeks, showing normal results. Growth scans were also performed at 29 weeks and 34 weeks, respectively, indicating normal growth. Within two weeks before delivery, she received two courses of dexamethasone, with a dosage of 20 mg taken once daily for four doses. In preparation for the delivery, tranexamic acid was administered on the morning of her scheduled C-section, with a dosage of 1 g taken three times a day for 48 hours, and she received 12 units of platelets both before and after the operation, with an additional 6 units administered during the operation. However, her platelet count remained persistently low despite receiving proper treatment and multiple platelet transfusions. Eventually, the patient underwent a C-section due to a breech presentation, resulting in an estimated blood loss of 900 ml. The fetal weight at birth was recorded as 2.520 kg, and the APGAR score was 5 at one minute and 9 at five minutes after birth. The patient was followed for weeks after a c-section, and her surgical wound has healed well with no complaints. She will be receiving ongoing follow-up care from both hematology and hepatology specialists and will also be provided with appropriate instructions for emergency room (ER) visits.

During her previous pregnancy, which ended at the 37th gestational week, the patient experienced significant changes in her hematological parameters. Her hemoglobin level before pregnancy measured 13.2 g/L. However, her hemoglobin level improved to 110 g/L during pregnancy. Conversely, her platelet count showed a decline from 42 × 10^9^/L before pregnancy to 29 × 10^9^/L during pregnancy. The patient was on nadolol at 80 mg/kg/day, prednisolone at 5 mg/kg/day, and azathioprine at 50 mg/kg/day. Additionally, she received a stress dose of hydrocortisone at 100 mg TID. A series of scans were performed to monitor the overall health and development of the fetus throughout pregnancy. An anatomy scan was conducted at 31 weeks of gestation, and a growth scan was performed at 36 weeks of gestational age, which yielded normal results. Before delivery, the patient received 6 units of platelets, and the baby was born through a vaginal delivery with a cephalic presentation and an estimated blood loss of 400 ml. The newborn weighed 2.820 kg and had APGAR scores of 9/9 at the first and fifth minutes, respectively, indicating a healthy condition.

## Discussion

AIH is a chronic liver condition characterized by immune system dysfunction, and its exact cause is unknown [1]. Numerous studies have extensively investigated the outcomes of AIH during pregnancy. A recent systematic review conducted in Portugal found that AIH flares occur in 7-33% of pregnancies and in 11-86% of cases during the postpartum period. Additionally, the occurrence of fetal growth restriction or newborns that are small for gestational age appears to be higher in patients with AIH. However, the incidence of fetal malformations is similar to that of the general population, and no correlation has been found with the medication used during pregnancy. Despite these risks, studies show that the rate of live births among AIH patients is similar to that of the general population [6]. Furthermore, another study reported by Westbrook et al. analyzed 81 pregnancies in AIH patients and found a live birth rate of 73% [12]. A retrospective study conducted in the Netherlands found that 6% of AIH patients experienced a relapse, while the relapse rate for postpartum episodes was 27%. However, pregnancies among AIH patients had a success rate of 98.5%, with 72% resulting in childbirth [8]. According to Schramn et al., approximately 20% of individuals with AIH experience a flare during pregnancy, with flares being more frequent during the postpartum period compared to during pregnancy. The exact immune mechanisms triggering these postpartum flares remain unclear [13]. It is worth mentioning that fetal mortality is also a concern in AIH pregnancies. Candia et al. discovered that 19% of women with AIH experienced fetal deaths before the 20th week of pregnancy, while Schramn et al. found a higher percentage of fetal mortality, reaching 24% in their analysis of a limited number of pregnant women with AIH [14].

In terms of medication use and efficacy during pregnancy in AIH, comprehensive studies have been conducted. In a retrospective study examining the management of AIH, 17 patients (37%) were identified as receiving immunosuppression monotherapy, with 14 patients on prednisone and 3 patients on azathioprine. Interestingly, no significant differences were observed between patients who experienced flares and those who did not. However, the study revealed that women who experienced a flare were more inclined to require combined therapy to sustain remission. Moreover, these women were found to be more susceptible to necessitating second-line treatment due to an inadequate response to the combined therapy. In addition, a single reported case of an unknown congenital malformation was observed in a pregnancy where azathioprine monotherapy was used. However, the outcome of the fetus exposed to mycophenolate mofetil (MMF) during the first two months of gestation was significantly positive [11].

Another case involved a 33-year-old female diagnosed with AIH type I who discontinued prednisolone treatment, resulting in elevated transaminase levels. Subsequently, budesonide at a dosage of 9 mg/day was initiated, proving to be effective in improving the patient's laboratory results. The patient opted not to pursue azathioprine, and approximately six months later she became pregnant. After five months of pregnancy, budesonide was gradually tapered and eventually discontinued upon confirmation of the pregnancy. At this stage, the patient's AIH condition appeared stable, and she successfully delivered a healthy child without experiencing any complications or disease flares [15].

In Turkey, a case report of a pregnant woman who had cirrhosis as a result of AIH was studied. Upon her hospitalization, it was found that she had severe thrombocytopenia with a platelet count of 27 × 10^3^/µl. As part of her treatment, she was prescribed a daily dose of 4 mg of prednisolone. The woman underwent a cesarean section at 36 weeks, 4 days after receiving immunoglobulin and platelet transfusions. The administration of low-molecular-weight heparin (LMWH) was temporarily halted until 12 hours after the operation. Unfortunately, she passed away due to a massive thromboembolism that occurred 24 hours after delivery [16].

In a study investigating the use of TPO-RAs for thrombocytopenia, a patient with severe chronic refractory ITP experienced successful treatment with Romiplostim during her first pregnancy [16]. Unintentionally, a second pregnancy occurred while she was receiving Eltrombopag. Despite this, she decided to continue taking eltrombopag for the entire duration of the pregnancy, which lasted 39 weeks, as she was determined not to interrupt the pregnancy. Remarkably, she gave birth to a healthy baby at full term, weighing 2 kg. The median duration of exposure to Tpo-RAs during pregnancy was 4.4 weeks, ranging from 1 to 39. Also In 17 pregnancies, thrombopoietin receptor agonists (Tpo-RAs) were initiated beyond week 32 of gestation to prepare for delivery. This decision was based on a platelet count of ≤ 20 × 10^9^/L in 10 cases (58%). Among the patients, four had preexisting chronic immune thrombocytopenic purpura (ITP) and were already receiving Tpo-RAs when they became pregnant, while in three cases, Tpo-RAs were initiated early in the third trimester due to symptomatic ITP that did not respond to standard therapy. The median platelet count at the time of Tpo-RA initiation was 10 × 10^9^/L (range: 1 × 10^9^/L to 20 × 10^9^/L). Ten patients (77%) demonstrated an initial positive response. However, three patients with longstanding chronic ITP, including two who had undergone splenectomy, did not exhibit any response to Tpo-RAs. Among the 10 respondents, 7 received additional concurrent ITP medication, primarily corticosteroids. At the time of delivery, the median platelet count was 94 × 10^9^/L (range: 6 × 10^9^/L to 250 × 10^9^/L). A platelet count below 50 × 10^9^/L was observed in only 5 out of the 17 pregnancies (29%), and no cases of severe maternal bleeding were observed [17].

The randomized placebo-controlled trials ADAPT-1 and ADAPT-2 demonstrated that Avatrombopag, a thrombopoietin receptor agonist, significantly increased platelet count and reduced the need for platelet transfusion in 435 patients with liver disease and severe thrombocytopenia who required an elective procedure. Notably, Avatrombopag effectively elevated platelet counts by 37,000 to 45,000/μL in patients with initial platelet counts ranging from 40,000 to 50,000/μL, with 87% of patients achieving a platelet count above 50,000/μL, which was the primary goal. Similarly, the L-PLUS-1 and L-PLUS-2 trials evaluated the efficacy of Lusutrombopag, another thrombopoietin receptor agonist, in 311 patients with liver disease and a platelet count less than 50,000/µL who required an invasive procedure. The trials demonstrated that Lusutrombopag significantly reduced the percentage of patients who required platelet transfusions compared to placebo (22-35% vs. 71-87%). Based on the positive results from the ADAPT and L-PLUS trials, both Avatrombopag and Lusutrombopag have received approval from the US Food and Drug Administration for use in augmenting the platelet count prior to elective procedures in patients with chronic liver disease-associated thrombocytopenia [18].

## Conclusions

In conclusion, pregnant women with autoimmune hepatitis require specialized antenatal care and close perinatal surveillance. A multidisciplinary approach is necessary to ensure comprehensive follow-up of these patients, and, with effective management, a successful pregnancy outcome is possible. However, clinicians should provide thorough counseling to patients about the potential complications associated with AIH even before conception to promote informed decision-making. Lastly, our study is limited due to the few reported cases, which can be attributed to the relative rarity of AIH. Future research studies and more cases need to be included to further explore the effects of medications and the disease course during pregnancy in patients with AIH.
